# In vitro evidence for the anti-staphylococcal activity of a cationic polymer compound–preliminary results

**DOI:** 10.1186/2047-2994-4-S1-I3

**Published:** 2015-06-16

**Authors:** P François, C Landelle, A Arndt, E-J Bonetti, A Renzoni, D Pittet, S Harbarth

**Affiliations:** 1 Dep. Medical Specialties, Univ Hosp of Geneva, Geneva, Switzerland; 2 Infection Control Program, Univ Hosp of Geneva, Geneva, Switzerland; 3 Prod Dev, B-Braun Medical AG, Sempach, Germany

## Introduction

In the context of a clinical trial, we observed a surprisingly high methicillin resistant *S. aureus* (MRSA) eradication rate of a placebo solution containing a cationic polymeric compound (CPC). The most active compound altering bacterial growth was identified by testing individually all molecules entering the composition of the commercial solution.

## Objectives

To assess the inhibitory capacity of a decolonisation solution and determine the *in vitro* time kill curves of Prontoderm^®^ (containing polyhexanide plus CPC) *versus* CPC alone and versus a control solution containing only excipients and emollients.

## Methods

Minimal inhibitory concentrations (MIC) of all compounds entering in the composition of a decolonization solution were assessed by a macro-method in liquid medium, on MRSA strains from the prevalent lineage isolated in our institution. A constant and calibrated inoculum of MRSA was exposed to adapted concentrations (4-fold MIC) of inhibitory compounds in liquid medium. Aliquots were sampled serially at time 15 to 240 min and diluted before plating on nutrient agar medium. Survival cells were enumerated after 20 h incubation at 37°C.

## Results

In addition to polyhexanide, the decolonisation solution contained another compound showing activity on MRSA growth. Polyhexanide was rapidly bactericidal for all tested MRSA strains; a rapid decrease of >5 logs in 15 min was generally observed. CPC showed also a significant effect on MRSA development. The killing was slower than that observed with polyhexanide but reached 4 to 5 logs after 2 h exposure.

## Conclusion

In addition to the active bactericidal compound, decolonization solution contains additives altering bacterial growth. The choice of a reliable placebo solution for comparison purposes in clinical trials should be adapted. Susceptibility of other relevant bacterial species, particularly multi-resistant pathogens, to the CPC should be evaluated.

## Disclosure of interest

P. François: None declared, C. Landelle: None declared, A. Arndt Employee of: B-Braun Medical AG, E.-J. Bonetti: None declared, A. Renzoni: None declared, D. Pittet: None declared, S. Harbarth Grant/Research support from: B-Braun Medical AG.

